# Discovery of high affinity inhibitors of *Leishmania donovani N*-myristoyltransferase†
†Electronic supplementary information (ESI) available. The coordinates and structure factor files have been deposited in the Protein Data Bank under the accession codes 5A27 and 5A28. See DOI: 10.1039/c5md00241a



**DOI:** 10.1039/c5md00241a

**Published:** 2015-08-19

**Authors:** Mark D. Rackham, Zhiyong Yu, James A. Brannigan, William P. Heal, Daniel Paape, K. Victoria Barker, Anthony J. Wilkinson, Deborah F. Smith, Robin J. Leatherbarrow, Edward W. Tate

**Affiliations:** a Department of Chemistry , Imperial College London , South Kensington Campus , London , SW7 2AZ , UK . Email: e.tate@imperial.ac.uk ; Tel: +44 (0) 2075 943752; b Structural Biology Laboratory , Department of Chemistry , University of York , York , YO10 5DD , UK; c Department of Biology , University of York , York , YO10 5DD , UK

## Abstract

*N*-Myristoyltransferase (NMT) is a potential drug target in *Leishmania* parasites. Scaffold-hopping from published inhibitors yielded the serendipitous discovery of a chemotype selective for *Leishmania donovani* NMT; development led to high affinity inhibitors with excellent ligand efficiency. The binding mode was characterised by crystallography and provides a structural rationale for selectivity.

## Abstract 2

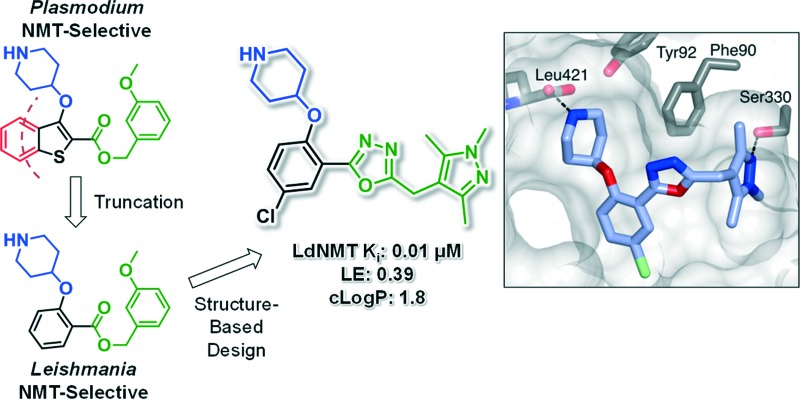
Truncation converted a *Plasmodium N*-myristoyltransferase inhibitor into a *Leishmania*-selective series, leading to a potent *L. donovani* NMT inhibitor through structure-guided design.

## Introduction

Leishmaniases are a spectrum of neglected tropical diseases resulting from the infection with parasites of the genus *Leishmania* spp., transmitted by the bite of the female *Phlebotomine* sandfly to the human host. The disease manifests in one of three forms (cutaneous, mucocutaneous and visceral)^1^ and is responsible for an estimated 60 000 deaths and in excess of 500 000 new cases per annum.^2^ The majority of fatalities stem from visceral leishmaniasis, which is predominately the result of infection by *Leishmania donovani* (Ld).^3^ As with many neglected diseases, the leishmaniases are most prevalent in developing and newly industrialized nations^4^ and although treatments exist, the majority suffer from significant levels of resistance and toxicity.^5^ *Leishmania* spp. infections remain challenging to treat chemotherapeutically; many currently marketed drugs display EC_50_ values in the low μM range in *in vitro* models,^5,6^ orders of magnitude higher than the potency typically targeted in an anti-infective drug discovery project. In order to combat the continued evolution of the parasite in developing resistance, new chemotherapies functioning by novel mechanisms of action are essential.

*N*-Myristoyltransferase (NMT) is a ubiquitous eukaryotic enzyme responsible for the attachment of the long chain fatty acid myristate to the N-terminus of substrate proteins. This modification is crucial in many biological processes,^7–10^ and NMT has been implicated as a highly promising drug target in fungal^11^ and parasitic infections,^12–14^ as recently demonstrated in human malaria parasites.^15^ In the specific context of leishmaniasis, NMT has been validated genetically in *Leishmania major* (Lm),^16^ and through chemical proteomic approaches in Ld,^17^ and selective small molecule inhibitors of this drug target are highly desirable.

## Results and discussion

**Fig. 1:**
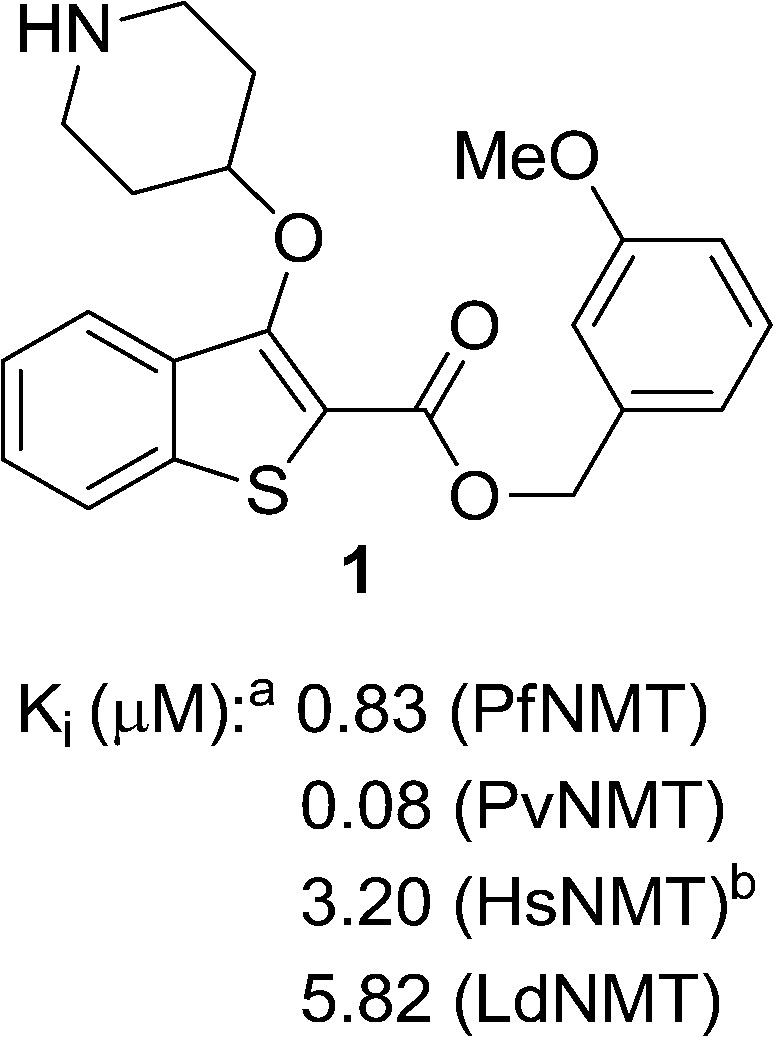
NMT affinity profile for 2,3-substituted benzo[*b*]thiophene **1**: ^a^Enzyme apparent *K*_i_ values are calculated from the IC_50_ values using the Cheng–Prusoff equation (see ESI†) to enable cross-comparisons. IC_50_ values were the mean of two or more independent determinations. Standard deviation is within 20% of reported IC_50_. ^b^HsNMT affinities reported in this work refer to HsNMT1; no significant difference in inhibition was observed between HsNMT1 and HsNMT2 isoforms.^18^

We have previously reported the discovery of LdNMT inhibitors by high-throughput screening,^18–21^ as well as the discovery of *Plasmodium falciparum* (Pf) and *Plasmodium vivax* (Pv) NMT inhibitors by both high-throughput screening^22^ and piggy-back/lead-hopping approaches.^23–25^ Throughout this work, all compounds were routinely screened against all three parasitic enzymes (LdNMT, PfNMT and PvNMT) and *Homo sapiens* (Hs) NMT, where selectivity over the human orthologue was desired to minimise any potential toxicity from modulating endogenous myristoylation. 2,3-Substituted benzo[*b*]thiophenes were discovered as ligand-efficient inhibitors of PfNMT, which incidentally displayed selectivity over LdNMT (Fig. 1).^25^

**Scheme 1:**
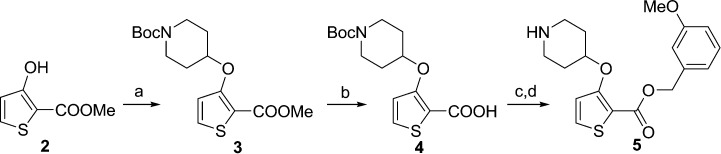
Synthesis of truncated inhibitor **5**. Reagents and conditions: (a) 1-Boc-4-piperidinol, DIAD, PPh_3_. THF, rt, 4 h, 94%; (b) NaOH, MeOH/H_2_O, 50 °C, 2 h, 90%; (c) (3-methoxyphenyl)methanol, EDCI, HOBt, DIPEA, MeCN, rt, 18 h; (d) 10% TFA in DCM (v/v), rt, 2 h, 9% over 2 steps.

During studies to reduce the molecular weight and lipophilicity of **1** in the further development of the series against plasmodial NMT, the bicyclic benzo[*b*]thiophene of **1** was truncated to a monocyclic scaffold (Scheme 1).

The affinity spectrum of compound **5** is strikingly distinct from that observed with benzo[*b*]thiophene **1** (Table 1). The activity against human and both *Plasmodium* enzymes drops by almost two orders of magnitude, whereas the affinity against LdNMT improves 8-fold, a significant improvement when considering the loss of 4 heavy atoms and attendant reduction in lipophilicity.

**Table 1:** Enzyme affinity and parasitic LE for mono- and bicyclic analogues **1**, **5** and **6**

No.	Structure	clog *P* ^*a*^	PfNMT		PvNMT		LdNMT		HsNMT *K*_i_ (μM)
			*K*_i_ (μM)	LE^*b*^	*K*_i_ (μM)	LE	*K*_i_ (μM)	LE	
**1**	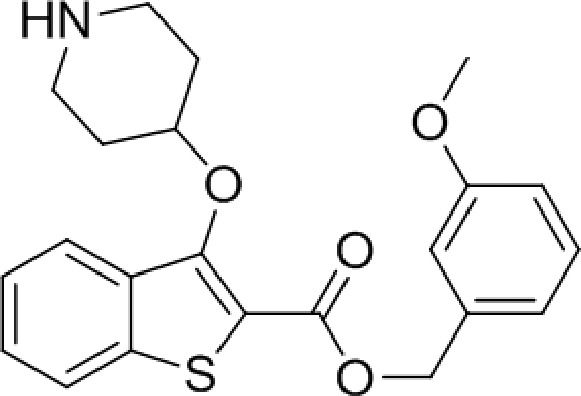	4.0	0.83	0.30	0.08	0.35	5.82	0.28	3.20
**5**	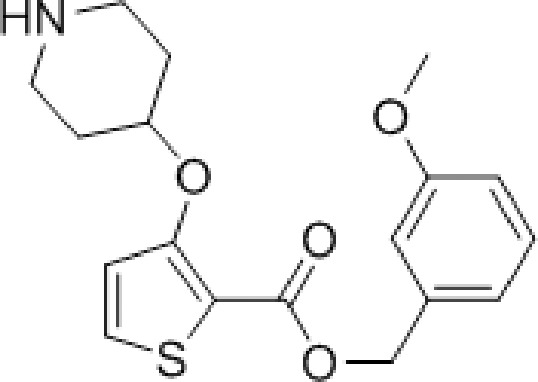	2.9	>100	—	6.50	0.30	0.73	0.35	>100
**6**	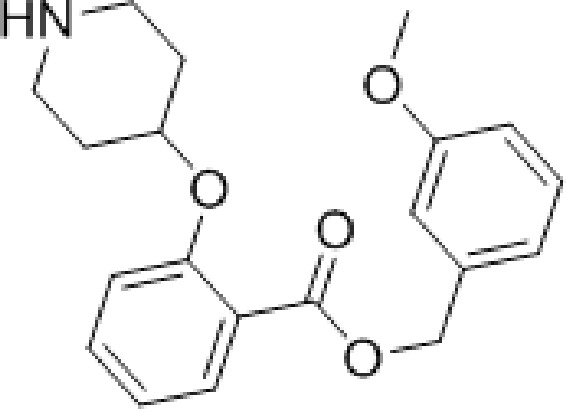	3.0	6.00	0.29	2.42	0.31	0.28	0.36	>100

^*a*^clog *P* values calculated with ChemAxon.

^*b*^LE = [–log(*K*_i_)](1.374)/(no. of heavy atoms).

Based on this affinity profile and structural analysis of the binding mode of **1** in PvNMT,^25^ it became clear that the monocyclic thiophene scaffold was unlikely to be a suitable chemotype for *Plasmodium* NMT inhibitors. However, **5** displays a very promising profile as a LdNMT inhibitor with excellent selectivity and LE, as well as sub-μM enzyme affinity. **5** was selected for further development, with the aim of discovering a new lead series for LdNMT inhibitors.

Although heterocycles are often preferred as bioisosteres for a phenyl ring, thiophene has been associated with cytochrome P450 inhibition and generally low “developability” during optimisation.^26^ Replacing the thiophene with a phenyl was deemed preferable, and provided a cost-effective synthetic route and versatility to variations around the scaffold. Pleasingly, this modification from thiophene to phenyl produced a further 3-fold improvement in enzyme affinity, whilst maintaining physicochemical properties and selectivity (Table 1). Interestingly, compounds with a phenyl scaffold proved amenable for development into high affinity *Plasmodium* NMT inhibitors – this work shall be reported elsewhere.^27^

**Scheme 2:**
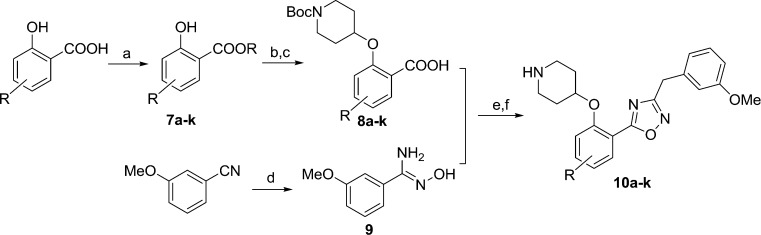
Synthesis of oxadiazole derivatives **10a–k**. Reagents: (a) BnBr, K_2_CO_3_, DMF, rt, 2 h, 72–89%; (b) DIAD, PPh_3_, *N*-Boc-4-OH piperidine, THF, rt, 4 h; (c) NaOH, MeOH/H_2_O, 50 °C, 2 h, 80–95% over two steps; (d) NH_2_OH, EtOH, 80 °C, 6 h, 98%; (e) i). EDCI, HOBt, **9**, DIPEA, CH_3_CN, rt, 4 h; ii). 0.5 N NaOH, rt, 0.5 h; (f) 10% TFA in DCM, rt, 2 h, 20–60% over 2 steps.

Compound **6** has a promising affinity profile, but further development of the series would require removal of the potentially labile ester moiety. We have previously reported the successful replacement of the ester moiety with an oxadiazole in related series;^24,28^ the same bioisosteric replacement was implemented in the present series, with the aim of producing a drug-like series for further development. The relatively straightforward synthetic scheme (Scheme 2) enabled facile synthesis of a variety of analogues, thereby allowing investigation of the preferred substituents on this ring system.

Moving from the ester linker in **6** to oxadiazole in **10a** produced a ~4-fold improvement in enzyme affinity alongside removal of the ester moiety. This compound displays measurable affinity against HsNMT but possesses selectivity of 20-fold in favour of the parasite enzyme, constituting a highly promising scaffold from which to investigate substituent effects on the phenyl ring (Table 2).

**Table 2:** Enzyme affinity and cellular potency for inhibitors based on a phenyl scaffold

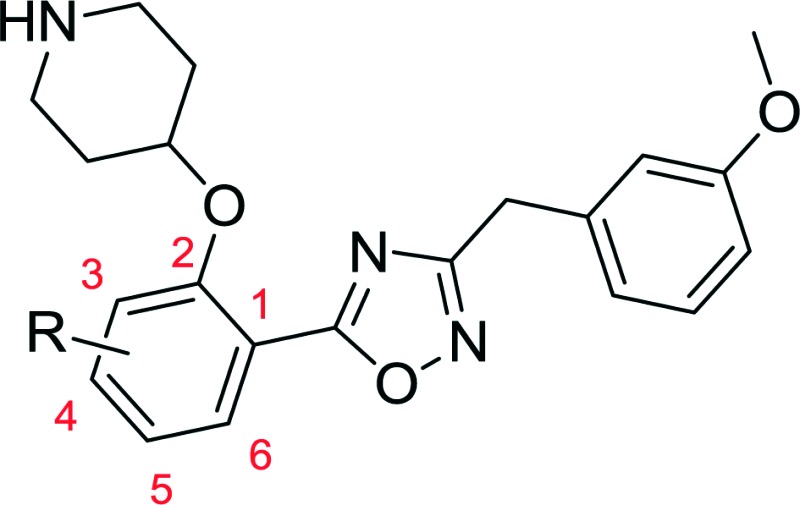							
No.	R	clog *P* ^*a*^	LdNMT		HsNMT *K*_i_ (μM)	Ld EC_50_ ^*c*^ (μM)	Macrophage LD_50_ ^*d*^ (μM)
			*K*_i_ (μM)	LE^*b*^			
**10a** ^*e*^	H	3.4	0.06	0.37	1.24	>30	—
**10b**	3-OMe	3.2	4.02	0.26	7.23	—	—
**10c**	3-Me	3.9	1.43	0.29	7.44	—	—
**10d**	4-Me	3.9	5.18	0.26	4.64	—	—
**10e** ^*e*^	4-OMe	3.2	3.04	0.26	0.86	—	—
**10f** ^*e*^	4-Cl	4.0	0.36	0.32	0.45	—	—
**10g** ^*e*^	4-F	3.5	0.03	0.37	0.59	10.2	29.5
**10h**	4-Br	4.2	0.68	0.30	0.26	—	—
**10i**	5-Me	3.9	0.24	0.32	0.76	—	—
**10j**	5-Cl	4.0	0.02	0.38	0.07	6.7	18.0
**10k**	5-OMe	3.2	0.05	0.35	0.76	13.5	27.9

No.	Structure	clog *P*	LdNMT		HsNMT *K*_i_ (μM)	Ld EC_50_ (μM)	Macrophage LD_50_ (μM)
			*K*_i_ (μM)	LE			
**13**	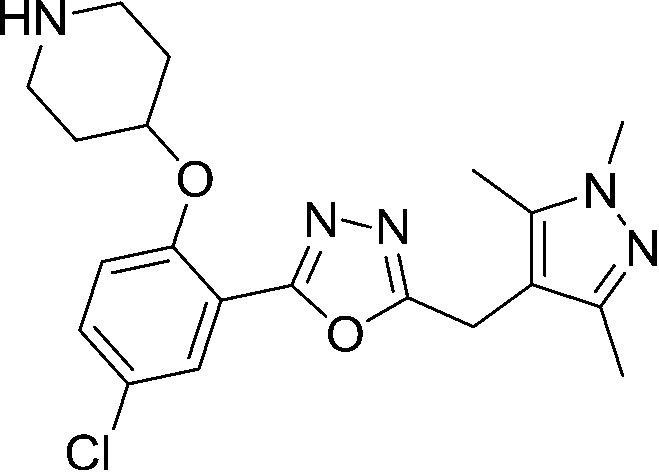	1.8	0.01	0.39	0.02	>50	>90

^*a*^clog *P* values calculated with ChemAxon.

^*b*^LE = [–log(Ki)] × 1.374/(no. of heavy atoms).

^*c*^ *Ex vivo L. donovani* amastigotes.^19^

^*d*^LD_50_ is an indication of general toxicity of a compound.

^*e*^This compound is described in Yu *et al.* ^27^

Comparison of **10c**, **10d** and **10i** shows the effect of scanning a methyl substituent around the 3-, 4- and 5-positions of the scaffold. In all cases this variation is less active than the unsubstituted parent **10a**, although among these three analogues the 5-substituted **10i** displays the highest affinity. This pattern repeats when comparing the methoxy-substituted **10b**, **10e** and **10k**, indicating that the 5-position is preferred for substitution. 5-OMe (**10k**) provides improved potency over 5-Me (**10i**), and this trend continues on moving to the 5-Cl substituent, identifying **10j** as a high affinity LdNMT inhibitor with a *K*_i_ of 0.02 μM.

The binding mode of **10j** to *Leishmania major* NMT (98% sequence identity to LdNMT with closely superimposable ligand binding sites)^14,29^ was confirmed by determination of the crystal structure of the ternary complex of LmNMT, myrisotyl-CoA and **10j** (PDB ID: ; 5a27). The inhibitor binds in the expected site adjacent to the enzyme's C-terminal carboxylate to which its piperidine forms an ion pairing interaction. This and other key interactions are conserved in a related series in PvNMT.^24^ Surprisingly, in addition to the observed interactions between the protein and the ligand, the crystal structure revealed a ring-opened derivative of the 1,2,4-oxadiazole, rather than the desired heterocycle (see Fig. S1 and S2†). However, proton NMR spectroscopy and high resolution mass spectrometry confirmed that the desired oxadiazole as the synthetic product, furthermore no degradation of **10j** was observed when this molecule was incubated at 37 °C over a wide pH range (pH 1–10, data not shown) over a period of 24 hours. This ‘ring opening’ phenomenon has been observed in related compounds within the program,^27^ although to our knowledge, it has not been reported elsewhere. The origin of the ring-opened species observed in the crystals remains unclear, and further investigation is underway to determine the cause of this discrepancy (such as potential radiation damage). As characterisation of the compounds was consistent with the proposed oxadiazoles, the development of the series was continued.

A selection of compounds with LdNMT *K*_i_ < 0.1 μM (**10a**, **10g**, **10j**, **10k**) were tested against *ex vivo* amastigotes, the clinically-relevant life cycle stage of the parasite, to determine the ability of these molecules to inhibit parasite growth. These molecules displayed a narrow (within 3-fold) therapeutic window against the macrophage host *in vitro*, and it was unclear whether the observed anti-parasitic activity is a result of NMT inhibition or non-specific toxicity. Given the relatively high clog *P* of the tested compounds (>3.5), it was hypothesised that reducing lipophilicity would lead to reduced macrophage toxicity.^30^ We therefore elected to build on our previous success in targeting the S319 pocket through polar heterocyclic replacement of the 3-methoxyphenyl, with the aim of producing a high affinity LdNMT inhibitor with reduced lipophilicity.^28^

**Scheme 3:**
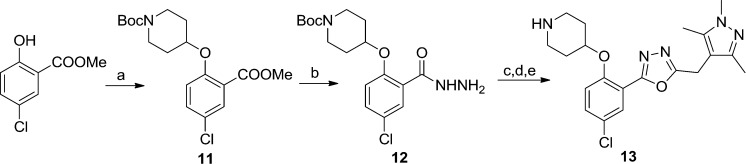
Synthesis of pyrazole derivative **13**. Reagents and conditions: (a) 1-Boc-4-piperidinol, DIAD, PPh_3_. THF, rt, 4 h, 46%; (b) hydrazine monohydrate, EtOH, reflux, 18 h, quantitative; (c) EDCI, HOBt, 2-(trimethyl-1*H*-pyrazol-4-yl)acetic acid,^28^ DIPEA, THF : DMF (4 : 1, v/v), rt, 18 h; (d) TsCl, 1,2,2,6,6-pentamethylpiperidine, DCM, rt, 3 h; (e) 10% TFA in DCM (v/v), rt, 2 h, 40% over three steps.

**Fig. 2:**
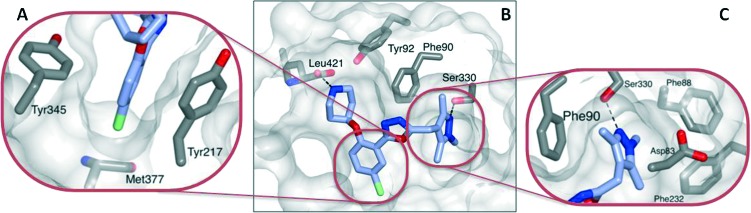
Crystal structure of **13** (blue) bound to LmNMT (grey). (A) The chloro-substituent is buried within a hydrophobic pocket, and appears to have good shape complementarity with the enzyme active site. (B) In addition to the shape-complementarity of the scaffold, the compound appears to form a similar binding mode to that previously observed. (C) The trimethylpyrazole motif forms both a hydrogen bonding interaction with Ser330 and a hydrophobic stacking interaction with Phe90.

Replacing the 1,2,4-oxadiazole with the more polar 1,3,4-regioisomer^31^ and utilising a 1,3,5-trimethylpyrazole motif to replace the hydrophobic methoxyphenyl moiety rendered compound **13** (Scheme 3). Encouragingly, **13** is roughly equipotent to **10j** yet is predicted to be two orders of magnitude less lipophilic, resulting in a highly promising compound. It should be noted that neither **10j** nor **13** display significant selectivity for LdNMT over HsNMT, highlighting a target for further development of the series. The crystal structure of **13** bound to LmNMT was solved (PDB ID: ; 5a28, Fig. 2), and this alternative oxadiazole isomer did not display the ring-opened analogue (Fig. S2†).

The basic piperidine moiety forms a polar interaction with the carboxylate of the C-terminal residue, Leu421 and a water-mediated interaction with Tyr92, mimicking the N-terminus of substrate peptides (Fig. 2B). In addition, the trimethyl pyrazole substituent forms π–π and polar interactions with Phe90 and Ser330 respectively (Fig. 2C), completing the interactions observed with previous NMT inhibitors. Of particular interest are the interactions around the inhibitor core; a qualitative assessment shows the 5-chlorine inserting into a hydrophobic pocket within the active site, and a lack of space around the 4-position of the scaffold reinforces the lack of activity of the larger substitutions in this position, in the absence of evidence for plasticity in the binding site (Fig. 2A). The rationale for the relative lack of activity of the 3-position substituents is less clear, but may be a result of placing a hydrophobic substituent in a water-filled cavity, or restriction of conformation of the piperidine moiety.

**Fig. 3:**
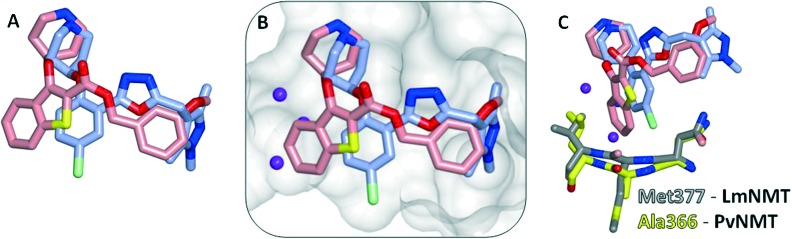
Comparison of the crystal structure of **1** (pink) bound to PvNMT (yellow) and **13** (blue) bound to LmNMT (grey). (A) Overlay of **1** in PvNMT and **13** in LdNMT, with the enzymes removed for clarity; the phenyl ring of the benzo[*b*]thiophene scaffold protrudes in the direction of potential 3- and 4-substitutions of the phenyl series. (B) The surface of the active site of LmNMT (grey) shows a constrained, water filled back pocket in the same position as that occupied by the benzo[*b*]thiophene. (C) Alanine to methionine substitution in LmNMT results in a more constrained pocket and a potential clash with the bulkier benzothiophene scaffold.

Interestingly, these conserved contacts of **1** and **13** are achieved with remarkably distinct trajectories through the binding site, and a comparison of the binding modes suggests a rationale for the large activity difference between PvNMT and LdNMT for **1** (Fig. 3).

The phenyl ring of the benzothiophene scaffold in **1** occupies a region of the PvNMT binding site that is not occupied by **13** in LmNMT (Fig. 3A), filling a volume occupied by two water molecules in LmNMT (Fig. 3B). Comparison of the residues around this pocket shows some significant differences, the largest of which is Ala366 in PvNMT substituted for Met377 in LmNMT (Fig. 3C). This shifts the protein backbone, and likely results in a more restricted pocket in LmNMT relative to PvNMT. The potential clash between the LmNMT backbone and the benzothiophene core of compound **1** provides a rationale for the reduced affinity of this molecule for LdNMT compared to PvNMT, and also an explanation for the activity loss in molecules carrying 4-substituents larger than fluorine (**10d–f**, **10h** *vs.* **10a**, Table 2).

Compound **13** was tested against both macrophages and Ld amastigotes. Pleasingly, **13** did not display any macrophage toxicity at the top concentration tested (90 μM), reinforcing the earlier hypothesis that reducing macrophage toxicity can be achieved by reducing lipophilicity. However, this molecule showed no observed effect against *ex vivo* amastigotes up to 50 μM.

## Conclusion

In summary, commencing from a member of a previously described series of PfNMT inhibitors, compound **5** was discovered as a highly ligand efficient inhibitor of LdNMT, and subsequent modification of the scaffold yielded the 5-chlorophenyl moiety as the optimum core. However, the compounds showed weak activity when tested against *ex vivo* amastigotes, and this appeared to correlate with macrophage toxicity; reduction of lipophilicity led to the discovery of compound **13**, a high affinity LdNMT inhibitor. The binding mode and selectivity could be rationalised by crystallography, and comparisons to previous structures. Whilst this molecule displayed no macrophage toxicity at all concentrations tested, it also failed to inhibit *L. donovani* amastigotes; the reasons for this lack of cellular activity remain unclear, but it is possible that this may be resolved through further optimisation of the amine basicity and lipophilicity balance in this series. Although NMT has great potential as a drug target in *Leishmania* parasites, these organisms are notoriously robust,^6^ and the physicochemical properties required for optimal engagement of intracellular parasite drug targets in the intra-macrophage amastigote stage remain poorly defined. Further investigation of the influences of the physicochemical properties of this series is currently underway, alongside the introduction of new NMT inhibitor chemotypes^18^ with the aim of chemically validating NMT as a drug target in *Leishmania donovani*.

## Notes

The authors declare no competing financial interest.
