# Supplementary material for: Discovery of high affinity inhibitors of Leishmania donovani N-myristoyltransferase
Source: Medchemcomm. 2015 Aug 19;6(10):1761–6. doi: 10.1039/c5md00241a (PMC4757855; doi:10.1039/c5md00241a)
Supplement: Supplementary file 1 [file MD-006-C5MD00241A-s001.pdf]

Electronic Supplementary Material (ESI) for MedChemComm.
This journal is © The Royal Society of Chemistry 2015

Discovery of high affinity inhibitors of *Leishmania donovani N*-myristoyltransferase

Mark D. Rackham,[1,∞,§] Zhiyong Yu,[1,∞,§] James A. Brannigan,[2] William P. Heal,[1,∞] Daniel Paape,[3,∞] K. Victoria Barker,[1,∞] Anthony J. Wilkinson,[2] Deborah F. Smith,[3] Robin J. Leatherbarrow, [1,∞] and Edward W. Tate[1*]

[1] Department of Chemistry, Imperial College London, South Kensington Campus, London, SW7 2AZ, U.K.

[2] Structural Biology Laboratory, Department of Chemistry, University of York, York, YO10 5DD, U.K.

[3]Department of Biology, University of York, York, YO10 5DD, U.K.

[∞]Current addresses: MDR - GlaxoSmithKline, Gunnels Wood Road, Stevenage, SG1 2NY, U.K.; ZY - International Discovery Service Unit, WuXi AppTec, Shanghai, 200131, China; WPH - Department of Chemistry, Kings College London, London SE1 1DB, U.K.; DP - Wellcome Trust Centre for Molecular Parasitology, Institute of Infection, Immunity and Inflammation, University of Glasgow, Glasgow, U. K.; KVB - Dehns, Aspect House, 84-87 Queens Road, Brighton, BN1 3XE, U.K.; RJL - Liverpool John Moores University, Egerton Court, 2 Rodney Street, Liverpool, L1 2UA, U.K.

SUPPORTING INFORMATION

CHEMICAL SYNTHESIS

General Methods. All chemicals were purchased from Sigma-Aldrich Ltd (Gillingham, UK), Acros Organics (Geel, Belgium) and Alfa Aesar (Heysham, UK) and used without further purification. Silica gel normal phase column chromatography was performed on an Isolera (Biotage, UK) automated apparatus with SNAP silica cartridges (Biotage, UK). Final compounds were purified on a Gilson semi-preparative Reverse Phase-HPLC system equipped with a HICHROM C_{18} Column (250 x 21.2 mm), #306 pumps and a Gilson UV/Vis detector, detecting at 220 nm. The mobile phase consisted of H_2O + 0.1 % Formic acid (solvent A) and MeOH + 0.1% Formic Acid (solvent B), with an elution method of 0-2 min 50% B, 2-30 min 50%-98% B, 30-32 min 98%, 32-32.5 min 2% B at a flow rate of 12 mL/min. Following purification, the organic solvent was removed under reduced pressure and the compounds dried by lyophilisation. The purity of title compounds was verified by RP-HPLC/MS on a Waters 2767 system equipped with a photodiode array and an ESI mass spectrometer using a XBridge C18 (5 μm, 4.6 mm × 100 mm) column, equipped with an XBridge C18 guard column (5 μm, 4.6 mm × 20 mm). The following elution method was used: Gradient of solvent A and solvent B (as above): 0-10 min 5-98% B, 10-12 min 98% B, 12-13 min 98 to 5% B, 13-17 min 5% B. Flow rate: 1.2 mL/min. Purity of tested compounds was ≥ 95%. ^{1}H spectra were recorded on 400 MHz Bruker AV instruments at room temperature and were referenced to residual solvent signals. Data are presented as follows: chemical shift in ppm, integration, multiplicity (app = apparent, br = broad, s = singlet, d = doublet, t = triplet, q = quartet, m = multiplet) and coupling constants in Hz. Mass spectra were obtained from the Mass Spectrometry Service of Department of Chemistry, Imperial College London.

SYNTHETIC PROCEDURES

Prototypical procedure for preparation of 4, 8a-k.

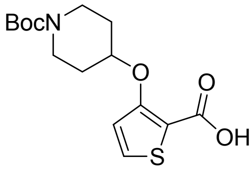

3-((1-(tert-Butoxycarbonyl)piperidin-4-yl)oxy)thiophene-2-carboxylic acid (4). To a stirred solution of **2** (150 mg, 0.95 mmol), *tert*-butyl-4-hydroxypiperidine-1-carboxylate (484 mg, 2.38 mmol) and triphenylphosphine (630 mg, 2.38 mmol) in anhydrous THF (4 mL), was added DIAD (459 µL, 2.38 mmol) dropwise at room temperature. The resulting mixture was stirred at room temperature for 4 hours, and then concentrated *in vacuo*. Purification by flash column chromatography (10g SNAP cartridge) furnished compound **3** as pale yellow oil. Compound **3** was then dissolved in a mixture of 4N NaOH (1.2 mL) and MeOH (5 mL) and the mixture was allowed to heat at 50 ºC for another 2 hours. After the reaction was complete, the mixture was partitioned between ethyl acetate (20 mL) and water (20 mL). The aqueous layer was then treated with 6N HCl to pH 2 and the neutral carboxylic acid was extracted into ethyl acetate (2 x 15 mL). After drying over $MgSO_4$, removal of organic solvent gave the title compound as an off-white solid (270 mg, 85% over two steps). ^{1}H NMR ($CDCl_3$, ppm) 7.64 (1H, d, J = 5.7Hz), 7.06 (1H, d, J = 5.7Hz), 4.74–4.66 (1H, m), 3.77–3.63 (2H, m), 3.53–3.41 (2H, m), 1.98–1.87 (2H, m), 1.86–1.76 (2H, m), 1.50 (9H, s).

Prototypical procedure for preparation of 5 and 6.

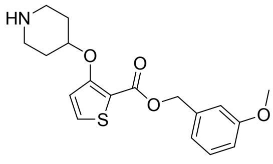

3-Methoxybenzyl-3-(piperidin-4-yloxy)-thiophene-2-carboxylate (5). A mixture of compound **4** (50 mg, 0.15 mmol), EDCI (33 mg, 0.17 mmol), HOBt (27 mg, 0.20 mmol) in dry acetonitrile (2 mL) was stirred at room temperature for 30 minutes, and then treated with (3-methoxyphenyl)methanol (23 mg, 0.16 mmol) and DIPEA (52 μL, 0.30 mmol). The resulting mixture was further stirred at room temperature for another 12 hours, and the solution was evaporated to dryness *in vacuo*. The residue was re-dissolved in ethyl acetate (20 mL) and washed with 0.5 M NaOH and brine sequentially. The organic layer was dried over anhydrous sodium sulphate and concentrated *in vacuo*, to produce the *N*-Boc precursor without further purification. The Boc-deprotection was carried out in DCM (1 mL) containing 10% TFA at room temperature for 2 h. After the reaction went to completion, the reaction mixture was evaporated under pressure to dryness, which was further purified by semi-preparative reverse phase HPLC to give the title compound as colourless oil (5 mg, 9% over two steps). ^{1}H NMR ($CDCl_3$, ppm) 8.43 (1H, brs) 7.46 (1H, d, *J*=5.5Hz), 7.31 (1H, app t, *J*=7.9Hz), 7.02 (1H, d, *J*=7.9Hz), 6.99-6.96 (1H,m), 6.88 (1H, dd, *J*=7.9, 2.3Hz), 6.82 (1H, d, *J*=5.5Hz), 5.28 (2H, s), 4.72-4.66 (1H, m), 3.82 (3H, s), 3.43-3.32 (2H, m), 3.21-3.13 (2H, m), 2.26-2.14 (2H, m), 2.14-2.04 (2H, m). ESI HRMS, found 348.1268 ($C_{18}H_{22}NO_4S$, $[M+H]^+$, requires 348.1270).

Prototypical procedure for preparation of 10a-k.

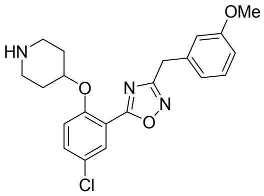

*4-(4-chloro-2-(3-[(3-methoxyphenyl)methyl]-1,2,4-oxadiazol-5-yl)phenoxy)piperidine (**10j**)*. A mixture of **8j** (54 mg, 0.15 mmol), EDCI (33 mg, 0.17 mmol), HOBt (27 mg, 0.20 mmol) in dry acetonitrile (3 mL) was stirred at room temperature for 30 minutes, and then treated with **9** (27 mg, 0.16 mmol) and DIPEA (52 μL, 0.30mmol). The resulting mixture was further stirred at room temperature for 4 hours. After the reaction went to completion, the solution was evaporated to dryness *in vacuo*. The residue was treated with 0.5 N NaOH (20 mL) and left for another 0.5 hr, followed by the extraction into ethyl acetate (2 x 10 mL). The combined organic layers were washed with brine, dried over $MgSO_4$, and concentrated under reduced pressure to give the *N*-Boc precursor without further purification. The above residue was re-dissolved in a solution of TFA (100 μL) and DCM (1 mL), then stirred at room temperature for 2 hours. The reaction mixture was evaporated under pressure to dryness, which was further purified by semi-preparative reverse phase HPLC to give the title compound as yellow oil (23 mg, 35% overall yield). ^{1}H-NMR (CD_3OD, ppm): 8.01 (1H, d, *J*=2.8 Hz) Ar-**H**, 7.58 (1H, dd, *J*=8.8, 2.8 Hz) Ar-**H**, 7.29-7.24 (2H, m) Ar-**H**, 6.98-6.89 (2H, m) Ar-**H**, 6.85 (1H, dd, *J*=8.8, 2.8 Hz) Ar-**H**, 5.10-4.98 (1H, m) R_2C**H-**OAr, 4.14 (2H, s) Ar-C**H$_2$**-Ar, 3.79 (3H, s) ArO-C**H$_3$**, 3.51-3.40 (2H, m) R-C**H$_2$**-R, 3.28-3.16 (2H, m) R-C**H$_2$**-R, 2.20-2.06 (4H, m) 2 x R-C**H$_2$**-R. ESI HRMS, found 400.1418 ($C_{21}H_{23}N_3O_3Cl$, $[M+H]^+$, requires 400.1428).

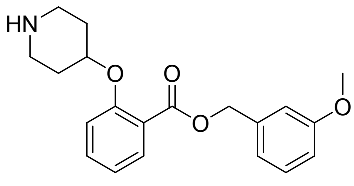

*(3-methoxyphenyl)methyl 2-(piperidin-4-yloxy)-benzoate (**6**).*

The synthetic route for compound **5** was followed to afford the title compound as an off-white solid in 15% yield. ^{1}H-NMR ($CDCl_3$, 400 MHz): δ 7.91 (d, J=7.2 Hz, 1H), 7.49 (app t, J=7.2 Hz, 1H), 7.33 (app t, J=7.2 Hz, 1H), 7.07-6.90 (m, 5H), 5.35 (s, 2H), 4.80 (brs, 1H), 3.84 (s, 3H), 3.48-3.35 (m,2H), 3.21-3.13 (m, 2H), 2.25-2.10 (m, 4H).

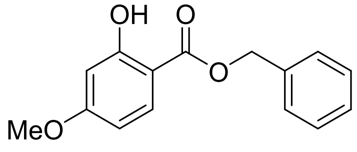

*benzyl 2-hydroxy-4-methoxybenzoate (7**e**).*A mixture of 2-hydroxy-4-methoxybenzoic acid (168 mg, 1 mmol), benzylbromide (125 µL, 1.05 mmol) and potassium carbonate (276 mg, 2 mmol) in DMF (2 mL) was stirred at room temperature for 2 hours. The reaction mixture was diluted with ethyl acetate (20 mL) and the organic phase washed with 20 mL water, then with brine (20 mL). The organic phase was dried over Na_2SO_4 and the organic solvent was removed under reduced pressure. The resulting residue was purified by column chromatography over silica gel to afford the title compound as a white solid (220 mg, yield: 86%). ^{1}H-NMR ($CDCl_3$, 400 MHz): δ 11.00 (s, 1H), 7.82 (d, J=7.6 Hz, 1H), 7.50-7.33 (m, 5H), 6.48 (dd, J=7.6, 2.4 Hz, 1H), 6.45 (d, J=2.4 Hz, 1H), 5.38 (s, 2H), 3.85 (s, 3H). All the other **7** series compounds were synthesised in an analogous fashion.

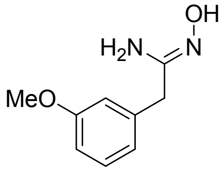

*(Z)-N'-hydroxy-2-(3-methoxyphenyl)ethenimidamide (**9**).* A solution of 2-(3-methoxyphenyl)acetonitrile (735 mg, 5 mmol) in MeOH (10 mL) was added 50% hydroxylamine (50% wt in H_2O, 1.25 mL, 20 mmol). The resulting mixture was kept at 65ºC for 6 hours, the solution was concentrated to afford the title compound as yellow oil (880 mg,

yield: 98%) without further purification. ^{1}H-NMR ($CDCl_3$, 400 MHz): δ 7.26 (app t, J=8.0 Hz, 1H), 6.90-6.80 (m, 2H), 4.52 (s, 1H), 3.82 (s, 3H), 3.45 (s, 2H).

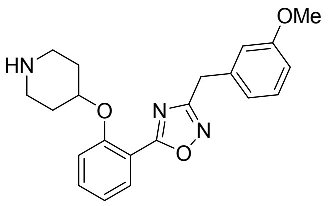

*3-(3-methoxybenzyl)-5-(2-(piperidin-4-yloxy)phenyl)-1,2,4-oxadiazole (**10a**)*. The synthetic route for compound **10j** was followed to afford the title compound as an off-white solid in 28% yield. ^{1}H-NMR ($CDCl_3$, 400 MHz): δ 8.09 (d, J=8.0 Hz, 1H), 7.55 (t, J=8.0 Hz, 1H), 7.28 (t, J=8.0 Hz, 2H), 7.14 (t, J=8.0 Hz, 1H), 7.06-6.96 (m, 3H), 6.84 (d, J=8.0 Hz, 1H), 4.91 (s, 1H), 4.18 (s, 2H), 3.55-3.50 (m, 2H), 3.22-3.19 (m, 2H), 2.27-2.24 (m, 2H), 2.16-2.13 (m, 2H). Calculated exact mass for the protonated molecule ($C_{21}H_{24}N_3O_3$): 366.1818; measured accurate mass (ESI): 366.1801.

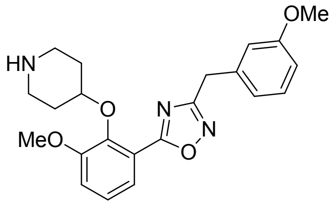

*4-(2-methoxy-6-{3-[(3-methoxyphenyl)methyl]-1,2,4-oxadiazol-5-yl}phenoxy)piperidine (**10b**)*. The synthetic route for compound **10j** was followed to afford the title compound as yellow oil in 46% yield. ^{1}H-NMR ($CDCl_3$, 400 MHz): δ 7.55 (dd, J=8.0, 1.6 Hz, 1H), 7.31 (dd, J=8.0, 1.6 Hz, 1H), 7.26-7.22 (m, 2H), 6.93-6.87 (m, 2H), 6.86-6.80 (m, 1H), 4.46-4.38 (m, 1H), 4.11 (s, 2H), 3.89 (s, 3H), 3.77 (s, 3H), 3.44-3.37 (m, 2H), 3.10-2.98 (m, 2H), 2.08-1.98 (m, 2H), 1.96-1.86 (m, 2H). ESI HRMS, found 396.1906 ($C_{22}H_{26}N_3O_4$, $[M+H]^+$, requires 396.1923).

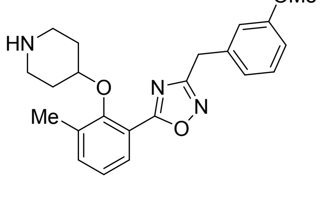

*4-(2-{3-[(3-methoxyphenyl)methyl]-1,2,4-oxadiazol-5-yl}-6-methylphenoxy)piperidine (**10c**)*. The synthetic route for compound **10j** was followed to

afford the title compound as yellow oil in 21% yield. ^{1}H-NMR (CD_3OD, 400 MHz): δ 7.83 (d, J=8.0 Hz, 1H), 7.53 (d, J=8.0 Hz, 1H), 7.28 (app t, J=8.0 Hz, 1H), 7.25 (app t, J=8.0 Hz, 1H), 6.99-6.93 (m, 2H), 6.87 (d, J=8.0 Hz, 1H), 4.14 (s, 2H), 4.06-3.97 (m, 1H), 3.81 (s, 3H), 3.31-3.24 (m, 2H), 2.39 (s, 3H), 2.05-1.94 (m, 2H), 1.91-1.79 (m, 4H). ESI HRMS, found 396.1906 ($C_{22}H_{26}N_3O_4$, $[M+H]^+$, requires 396.1923).

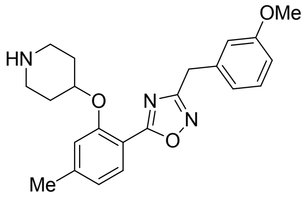

*4-(2-{3-[(3-methoxyphenyl)methyl]-1,2,4-oxadiazol-5-yl}-5-methylphenoxy)piperidine (**10d**).* The synthetic route for compound **10j** was followed to afford the title compound as a yellow oil in 30% yield. ^{1}H-NMR (CD_3OD, 400 MHz): δ 7.96 (d, J=8.0 Hz, 1H), 7.27 (app t, J=8.0 Hz, 1H), 6.98 (d, J=8.0 Hz, 1H), 6.95-6.90 (m, 2H), 6.83-6.79 (m, 2H), 4.89-4.82 (m, 1H), 4.14 (s, 2H), 3.81 (s, 3H), 3.52-3.41 (m, 2H), 3.22-3.11 (m, 2H), 2.42 (s, 3H), 2.29-2.05 (m, 4H). ESI HRMS, found 380.1968 ($C_{22}H_{26}N_3O_3$, $[M+H]^+$, requires 380.1974).

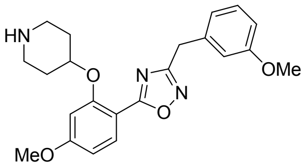

*5-(4-methoxy-2-(piperidin-4-yloxy)phenyl)-3-(3-methoxybenzyl)-1,2,4-oxadiazole (**10e**).* The synthetic route for compound **10j** was followed to afford the title compound as a yellow oil in 20% yield. ^{1}H-NMR ($CDCl_3$, 400 MHz): δ 8.04 (d, J=8.8 Hz, 1H), 7.27-7.22 (m, 1H), 6.99-6.81 (m, 3H), 6.64 (dd, J=8.8, 2.4 Hz, 1H), 6.52 (d, J=2.4 Hz, 1H), 4.85-4.78 (m, 1H), 4.13 (s, 2H), 3.88 (s, 3H), 3.81 (s, 3H), 3.47-3.36 (m, 2H), 3.22-3.11 (m, 2H), 2.28-2.02 (m, 4H). Calculated exact mass for the protonated molecule ($C_{22}H_{26}N_3O_4$): 396.1923; measured accurate mass (ESI): 396.1919.

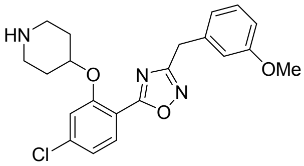

*5-(4-chloro-2-(piperidin-4-yloxy)phenyl)-3-(3-methoxybenzyl)-1,2,4-oxadiazole (**10f**).* The synthetic route for compound **10j** was followed to afford the title

compound as a yellow oil in 56% yield. ^{1}H-NMR (CD$_3$OD, 400 MHz): δ 8.04 (d, J=8.4 Hz, 1H), 7.39 (d, J=1.6 Hz, 1H), 7.26 (t, J=8.0 Hz, 1H), 7.19 (dd, J=8.4, 1.6 Hz, 1H), 6.97-6.88 (m, 2H), 6.84 (dd, J=8.0, 1.6 Hz, 1H), 5.05-4.98 (m, 1H), 4.13 (s, 2H), 3.79 (s, 3H), 3.52-3.41 (m, 2H), 3.28-3.18 (m, 2H), 2.20-2.08 (m, 4H). Calculated exact mass for the protonated molecule ($C_{21}H_{23}N_3O_3Cl$): 400.1428; measured accurate mass (ESI): 400.1428.

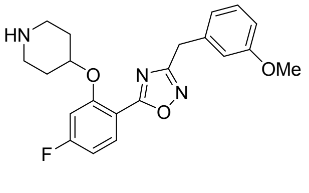

*5-(4-fluoro-2-(piperidin-4-yloxy)phenyl)-3-(3-methoxybenzyl)-1,2,4-oxadiazole (**10g**).* The synthetic route for compound **10j** was followed to afford the title compound as a yellow solid in 44% yield. ^{1}H-NMR (CD$_3$OD, 400 MHz): δ 8.11 (dd, J=8.4, 6.8 Hz, 1H), 7.26 (t, J=8.0 Hz, 1H), 7.16 (dd, J=6.8, 2.4 Hz, 1H), 6.97-6.89 (m, 3H), 6.84 (dd, J=8.4, 2.4 Hz, 1H), 5.03-4.98 (m, 1H), 4.13 (s, 2H), 3.79 (s, 3H), 3.53-3.42 (m, 2H), 3.29-3.19 (m, 2H), 2.22-2.09 (m, 4H). Calculated exact mass for the protonated molecule ($C_{21}H_{23}N_3O_3F$): 384.1723; measured accurate mass (ESI): 384.1723.

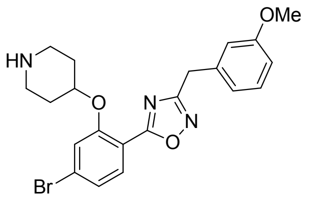

*4-(5-bromo-2-{3-[(3-methoxyphenyl)methyl]-1,2,4-oxadiazol-5-yl}phenoxy)piperidine (**10h**).* The synthetic route for compound **10j** was followed to afford the title compound as yellow oil in 60% yield. ^{1}H-NMR (CD$_3$OD, 400 MHz): δ 7.96 (d, J=8.4 Hz, 1H), 7.53 (d, J=1.6 Hz, 1H), 7.34 (dd, J=8.4, 1.6 Hz, 1H), 7.25 (app t, J=8.4 Hz, 1H), 6.97-6.88 (m, 2H), 6.84 (d, J=8.4 Hz, 1H), 5.05-4.98 (m, 1H), 4.13 (s, 2H), 3.79 (s, 3H), 3.52-3.41 (m, 2H), 3.28-3.19 (m, 2H), 2.20-2.09 (m, 4H). ESI HRMS, found 444.0919 ($C_{21}H_{23}N_3O_3Br$, $[M+H]^+$, requires 444.0923).

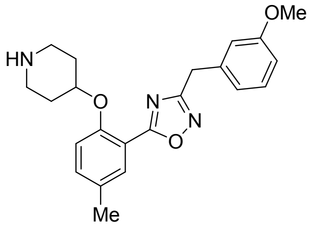

4-(2-{3-[(3-methoxyphenyl)methyl]-1,2,4-oxadiazol-5-yl}-4-methylphenoxy)piperidine (10i). The synthetic for compound **10j** was followed to afford the title compound as yellow oil in 48% yield. ^{1}H-NMR (CD_3OD, 400 MHz): δ 7.87 (d, J=2.0 Hz, 1H), 7.44 (dd, J=8.4, 2.0 Hz, 1H), 7.27 (app t, J=8.4 Hz, 1H), 7.19 (d, J=8.4 Hz, 1H), 6.99-6.92 (m, 2H), 6.90-6.83 (m, 1H), 5.02-4.93 (m, 1H), 4.14 (s, 2H), 3.81 (s, 3H), 3.52-3.44 (m, 2H), 3.26-3.17 (m, 2H), 2.37 (s, 3H), 2.17-2.10 (m, 4H). ESI HRMS, found 380.1961 ($C_{22}H_{26}N_3O_3$, $[M+H]^+$, requires 380.1974).

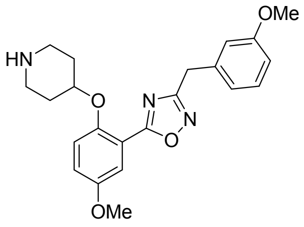

4-(4-methoxy-2-{3-[(3-methoxyphenyl)methyl]-1,2,4-oxadiazol-5-yl}phenoxy)piperidine (10k). The synthetic route for compound **10j** was followed to afford the title compound as yellow oil in 45% yield. ^{1}H-NMR (CD_3OD, 400 MHz): δ 7.56 (d, J=2.8 Hz, 1H), 7.26 (app t, J=8.0 Hz, 1H), 7.24-7.17 (m, 2H), 6.98-6.89 (m, 2H), 6.85 (dd, J=8.0, 2.8 Hz, 1H), 4.84-4.78 (m, 1H), 4.14 (s, 2H), 3.83 (s, 3H), 3.80 (s, 3H), 3.51-3.40 (m, 2H), 3.24-3.13 (m, 2H), 2.17-2.04 (m, 4H). ESI HRMS, found 396.1914 ($C_{22}H_{26}N_3O_4$, $[M+H]^+$, requires 380.1923).

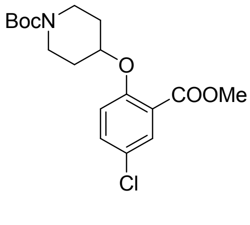

tert-butyl 4-[4-chloro-2-(methoxycarbonyl)phenoxy]piperidine-1-carboxylate (11). The first step to make compound **8a** was followed to afford the title compound as colourless oil (46% yield). ^{1}H NMR ($CDCl_3$, 400 MHz): δ 7.78 (d, J=2.7 Hz, 1H), 7.39 (dd, J=8.9, 2.7 Hz, 1H), 6.93 (d, J=8.9 Hz, 1H), 4.57 (tt, J=5.9, 3.7 Hz, 1H), 3.89 (s, 3H), 3.65-3.55 (m, 2H), 3.55-3.44 (m, 2H), 1.93-1.78 (m, 4H), 1.47 (s, 9H).

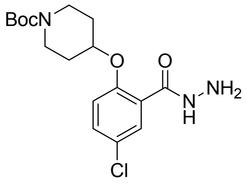

*tert-Butyl 4-(4-chloro-2-(hydrazinecarbonyl)phenoxy)piperidine-1-carboxylate (**12**).* To a solution of **11** (450 mg) in ethanol (3 mL) was added hydrazine monohydrate (600 μL) and reaction was allowed to stir at 78 °C for 18 h. Volatiles removed under reduced pressure and product recovered without further purification. ^{1}H NMR ($CDCl_3$, ppm) 8.83 (1H, brs), 8.10 (1H, d, *J*=2.8 Hz), 7.34 (1H, dd, *J*=8.8, 2.8 Hz), 6.91 (1H, d, *J*=8.8 Hz), 4.58 (1H, app tt, *J*=7.8, 3.7 Hz), 3.84-3.70 (2H, m), 3.27-3.20 (2H, m), 2.11-1.96 (2H, m), 1.82-1.70 (2H, m), 1.45 (9H, s).

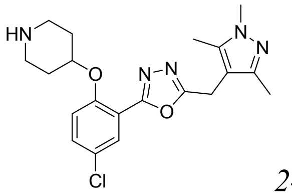

*2-(5-Chloro-2-(piperidin-4-yloxy)phenyl)-5-((1,3,5-trimethyl-1H-pyrazol-4-yl)methyl)-1,3,4-oxadiazole (**13**).* To a solution of **12** (66 mg, 0.18 mmol) in THF/DMF (4:1 *v*/*v*, 1.5 mL) was added HOBt (32 mg, 0.23 mmol), EDCI (38 mg, 0.20 mmol) and 2-(trimethyl-1H-pyrazol-4-yl)acetic acid (34 mg, 0.20 mmol).[1] Reaction mixture was allowed to stir at room temperature for 18 h, then diluted with 0.5 M NaOH (10 mL), followed by extraction with EtOAc (2 x 10 mL). Combined organic layers were washed with brine (20 mL), dried over sodium sulfate and concentrated under reduced pressure, yielding pale orange oil which was used without further purification (89 mg). The above orange oil (0.17 mmol) and 1,2,2,6,6-pentamethyl-piperidine (65 μL, 0.36 mmol) in dichloromethane (1 mL) was added TsCl (34 mg, 0.18 mmol) and the reaction mixture was allowed to stir at room temperature for 3 h. The reaction mixture was then diluted with a further 10 mL dichloromethane, washed with water (10 mL), 0.5 M NaOH (10 mL), brine (10 mL),

dried over magnesium sulfate and concentrated under reduced pressure. The resulting pale yellow oil was re-dissolved in a solution of DCM (1 mL) containing 10% TFA (*v/v*). The mixture was stirred at room temperature for 2 hours. The reaction mixture was evaporated under pressure to dryness, which was further purified by semi-preparative reverse phase HPLC to give the title compound a colourless oil (28 mg, 35% overall yield). ^{1}H NMR (MeOD, ppm) 8.52 (1H, brs) N**H**, 7.84 (1H, d, *J*=2.7 Hz) Ar**H**, 7.57 (1H, dd, *J*=9.0, 2.7 Hz) Ar**H**, 7.32 (1H, d, *J*=9.0 Hz) Ar**H**, 5.01-4.95 (1H, m) R_2C**H-**OAr, 4.12 (2H, s) Ar-C$\mathbf{H}_2$-Ar, 3.73 (3H, s) N-C$\mathbf{H}_3$, 3.52 (2H, ddd, *J*=14.0, 9.9, 4.7 Hz) R-C$\mathbf{H_2}$-R, 3.28-3.24 (2H, m) R-C$\mathbf{H_2}$-R, 2.30 (3H, s) Ar-C$\mathbf{H_3}$, 2.22 (3H, s) Ar-C$\mathbf{H_3}$, 2.16-2.02 (4H, m) 2 x R-C$\mathbf{H_2}$-R. ESI HRMS, found 402.1696 ($C_{20}H_{25}N_5O_2Cl$, $[M+H]^+$, requires 402.1697).

ASSAYS AND CRYSTALLOGRAPHY

Enzyme assay information. All IC_{50} determinations were carried out using a 7-diethylamine-3-(4'maleimidylphenyl)-4-methylcoumarin (CPM) fluorescence assay, as described by previously.[2–4] The HsNMT1, PfNMT and PvNMT assays are as described in the literature, for LdNMT the final enzyme concentration and peptide substrates are: [LdNMT]: 400 ng/mL; LdNMT Peptide Substrate: *Homo sapiens* p60^{src} (2-16) with sequence GSNKSKPKDASQRRR-NH_2 and the final concentration was 4.0 µM. IC_{50} of an inhibitor was calculated by a nonlinear regression analysis using GraFit 7.0.1 version (Erithacus Software Limited, UK).The values are the mean value of two determinations; standard deviation is within 20% of the IC_{50} unless otherwise specified.

K_i values were calculated from the experimentally determined IC_{50} values, the substrate concentration ([S]) and the Michaelis-Menten constant (K_m) as described by the Cheng-Prusoff equation:[5]

Equation 1: Cheng-Prusoff Equation for Determination of K_i from IC_{50}

$$K_i = \frac{IC_{50}}{1 + [S]/K_m}$$

For example, compound **5** had an experimentally determined IC_{50} of 0.82 ± 0.14 µM. The Michaelis Constant (K_m) was 32.7 µM and the substrate concentration was 4.0 µM, giving a K_i of 0.73 µM.

***L. donovani ex-vivo* amastigotes inhibition assay.** Assays were carried out as described by Paape and colleagues.[6] Briefly, Ld amastigotes (4 × 10^5/well of 96-well plate) were incubated with test compounds, with the concentrations ranging from 75 µM to 0.034 µM in 200 µL RPMI 1640 medium supplemented with 20% heat-inactivated FCS, 100 µM adenine, 20 mM 2-[*N*-morpholino]ethanesulphonic acid (pH 5.5), 5 µM hemin, 3 µM biopterin, 1 µM biotin, 100 U penicillin and 100 µg streptomycin. After 72 hour incubation at 26 ˚C, alamar blue (10% v/v) was added to each well and the resulting mixture was incubated for additional 4 hours prior to measuring the fluorescence (excitation wavelength was 544 nm and emission was recorded with a 590nm±10nm bandpass filter, POLARstar Optima). Parasites cultured in medium alone were used as a positive control and medium without cells containing corresponding concentration of a compound was used as a medium control. Growth inhibition at each concentration was calculated as % inhibition of normalized data = [1-(readout-medium)/(positive-medium)] x 100%. Assays were carried out in triplicate.

Macrophage toxicity test. Assays were carried out as described by Paape and colleagues.[6] Briefly, bone marrow-derived macrophage (BMDM, 4.2 × 10^4/well of 96-well plate) were incubated at the relevant concentrations of test compounds in DMEM medium containing 4% L929 cell and 10% FCS (fetal calf serum). Cells were

incubated at 37° C and 5% CO_2 for 72 hours. Aalamar blue (10% v/v) was added to each well and the resulting mixture was incubated for additional 4 hours prior to measuring the fluorescence as described above. Medium without cells containing the corresponding concentration of compound was used as a medium control. Assays were performed in duplicate.

Crystallography. Procedure for protein expression, purification, crystallization and data processing were described in detail in an earlier paper.[7] Data refinement can be found in Table S1.

Table S1. X-ray data collection and refinement statistics

**PDB accession code**	LmNMT-MyrCoA-**10j** **5a27**	LmNMT-MyrCoA-**13** **5a28**
Cell dimensions *a*, *b*, *c* (Å)	46.94, 90.59, 52.71	48.45, 92.16, 53.46
Cell angles α, β, γ (°)	90.0, 111.5, 90.0	90.0, 113.8, 90.0
Space Group	$P2_1$	$P2_1$
**Data collection**		
Beamline / Wavelength (Å)	DLS i04-1 / 0.9795	DLS i04-1 / 0.9200
Detector type	Pilatus CMOS	Pilatus CMOS
Images x oscillation (°)	400 x 0.5	1100 x 0.2
Resolution (Å)	22–1.37 (1.41–1.37)	49–1.48 (1.51–1.48)
R_{sym} (%) [b]	5.3 (40.5)	3.7 (68.3)
$I/\sigma I$	13.2 (2.2)	15.7 (1.6)
Completeness (%)	94.8 (65.5)	98.9 (99.1)
Redundancy	3.9 (2.7)	4.2 (4.2)
**Refinement**		
No. unique reflections	81394	70591
R_{work} / R_{free}[c]	14.6 / 18.2	20.1 / 24.5
No. atoms	4423	4018
Protein	3587	3563
Ligand	28	28
Co-factor	63	63
Water	706	363
B-factors (Å^2)		
All atoms	14.4	27.5
Protein	12.6	26.9
Ligand	18.3	20.7
Co-factor	8.9	18.6
Water	26.0	34.2
R.m.s.deviations [d]		
Bond lengths (Å)	0.026	0.026
Bond angles (°)	2.482	2.503

[a] Highest resolution shell is shown in parentheses.

[b] $R_{sym} = \Sigma_{\mathbf{h}}\Sigma_l |I_{\mathbf{h}l} - <I_{\mathbf{h}}>| / \Sigma_{\mathbf{h}}\Sigma_l <I_{\mathbf{h}}>$, where I_l is the lth observation of reflection h and $<I_{\mathbf{h}}>$ is the weighted average intensity for all observations l of reflection h.

[c] $R_{cryst} = \sum||F_o| - |F_c||/\sum|F_o|$ where F_o and F_c are the observed and calculated structure factor amplitudes, respectively. R_{free} is the R_{cryst} calculated with 5% of the reflections omitted from refinement.

[d] Root-mean-square deviation of bond lengths or bond angles from ideal geometry.

SUPPLEMENTARY FIGURES

Figure S1

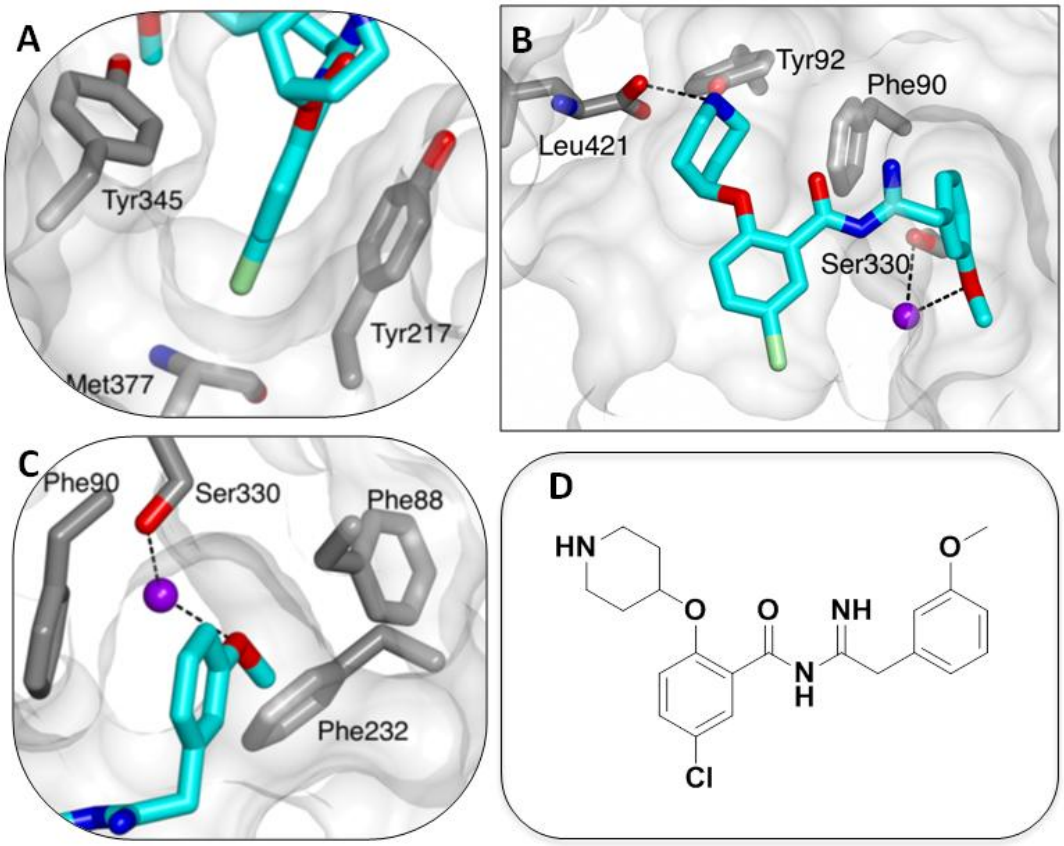

Figure S1: Crystal structure of **10j** (blue) bound to LmNMT (grey) – PDB ID: 5a27. (A) The chloro-substituent is buried within a hydrophobic pocket. (B) In addition to the shape-complementarity of the scaffold, the compound appears to form the polar interactions with the enzyme C-terminal residue, Leu421, and a water-bridged hydrogen bond to Ser330. Unexpectedly, the structure appears to show a ring-opened derivative of the 1,2,4-oxadiazole, rather than the expected heterocycle. (C) The 3-methoxyphenyl motif forms both a polar interaction with Ser330 and a π-stacking interaction with Phe90. (D) **10j** was modelled as the *N*-(1-iminoalkyl) tautomer shown, although it is recognised that other tautomers are likely to exist.

Figure S2

A B C D

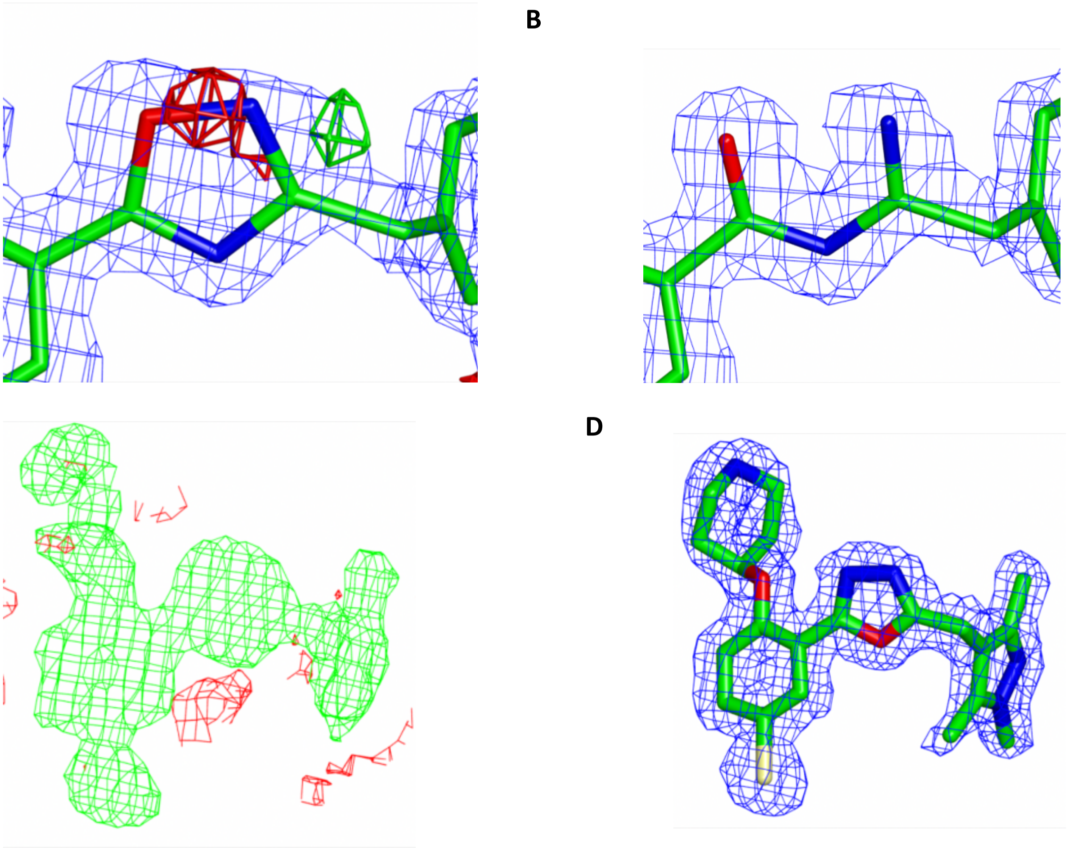

Figure S2: Electron density maps relating to compounds **10j** and **13** bound to LmNMT. (A) Electron density maps calculated during early stages of refinement revealed regions of negative (red) and positive (green) density in the difference map (mF_O-DF_C contoured at a level of 3σ) which are inconsistent with the presence of a planar oxadiazole ring in compound **10j**. (B) These discrepancies did not appear when a ring-open form was used as the modelled ligand and the final, refined electron density map (blue, 2mF_O-DF_C) around the ligand contoured at a level of 1σ is shown. (C) An initial 'omit' map calculated in the absence of ligand **13** shows clear density in the difference map for an associated molecule. (D) The final, refined electron density map is entirely consistent with a planar ring in bound compound **13**. Ligands are represented as cylinders and coloured by atom (carbon, green; oxygen, red; nitrogen, blue and fluorine, lemon). Electron density is shown as 'chicken-wire' and displayed using the program CCP4mg.

References

1 M. D. Rackham, J. A. Brannigan, K. Rangachari, S. Meister, A. J. Wilkinson, A. A. Holder, R. J. Leatherbarrow and E. W. Tate, *J. Med. Chem.*, 2014, **57**, 2773–2788.

2 V. Goncalves, J. A. Brannigan, D. Whalley, K. H. Ansell, B. Saxty, A. A. Holder, A. J. Wilkinson, E. W. Tate and R. J. Leatherbarrow, *J. Med. Chem.*, 2012, **55**, 3578–82.

3 M. D. Rackham, J. A. Brannigan, D. K. Moss, Z. Yu, A. J. Wilkinson, A. A. Holder, E. W. Tate and R. J. Leatherbarrow, *J. Med. Chem.*, 2013, **56**, 371–375.

4 V. Goncalves, J. A. Brannigan, E. Thinon, T. O. Olaleye, R. Serwa, S. Lanzarone, A. J. Wilkinson, E. W. Tate and R. J. Leatherbarrow, *Anal. Biochem.*, 2012, **421**, 342–4.

5 Y. Cheng and W. H. Prusoff, *Biochem. Pharmacol.*, 1973, **22**, 3099–108.

6 D. Paape, A. S. Bell, W. P. Heal, J. A. Hutton, R. J. Leatherbarrow, E. W. Tate and D. F. Smith, *PLoS Negl. Trop. Dis.*, 2014, **8**, e3363.

7 Z. Yu, J. A. Brannigan, D. K. Moss, A. M. Brzozowski, A. J. Wilkinson, A. A. Holder, E. W. Tate and R. J. Leatherbarrow, *J. Med. Chem.*, 2012, **55**, 8879–90.
